# Under threat by popular vote: German-speaking immigrants’ affect and cognitions following the Swiss vote against mass immigration

**DOI:** 10.1371/journal.pone.0175896

**Published:** 2017-04-13

**Authors:** Selma Carolin Rudert, Stefan Janke, Rainer Greifeneder

**Affiliations:** 1 Department of Psychology, University of Basel, Basel, Switzerland; 2 Department of Psychology, University of Mannheim, Mannheim, Germany; Centre Hospitalier Universitaire Vaudois, SWITZERLAND

## Abstract

A popular initiative in support of regulating future immigration to Switzerland was accepted by the electorate in 2014. Assuming that the initiative acted as an exclusionary threat for current immigrants of Switzerland, we conducted an online survey among a sample of highly-skilled German-speaking immigrants (“expats”). Participants reported having experienced negative affect following the vote. Moreover, having a more left-wing orientation, living in a political constituency that had voted pro-regulation and having proportionally few Swiss friends positively predicted negative affect following the vote. Negative affect was associated with a reported negative change in one’s attitudes towards Switzerland, increased considerations to leave the country, and impaired satisfaction with life. In sum, the results suggest that a powerful exclusionary threat such as a national vote may be experienced as distressful by highly-skilled immigrants currently living in the country.

## Introduction

On February 9^th^, 2014, the Swiss popular initiative “Against mass immigration”was put to the vote and accepted by the electorate. Popular initiatives are a means of direct democracy in Switzerland and allow the Swiss people to suggest or change laws directly via nation-wide votes. The aim of this particular initiative was to limit immigration to Switzerland through quotas and thereby restrict the number of immigrants moving to Switzerland each year [1]. The initiative was strongly debated, but eventually accepted by a narrow majority of the electorate (50.3 percent) and of the cantons (member states of the federal state of Switzerland; 17 out of 26; [2]), which is necessary for a nation-wide initiative to succeed (for more details on Swiss direct democracy and popular votes, see [3]).

Against the background of laboratory research on social exclusion ([4–15]; for further details, see below), such a national vote can likely act as an exclusionary threat to immigrants. Here we investigate the distress and threat that highly-skilled immigrants experienced following the vote. Immigrants formed a total of 24.3 percent of Switzerland’s total population in 2014; [16]), with German-speaking immigrants representing one of Switzerland’s largest immigrant groups [17, 18]. In the present study, we focus on the reactions of a sample of highly-skilled German-speaking immigrants (so-called “expats”) who already live in Switzerland but do not have Swiss citizenship. While direct conclusions are refined to this group, focusing on this group is of particular interest for at least two reasons: First, because the initiative aims for the regulation of *future* immigration, the result of the vote neither directly forces current immigrants to leave the country, nor does it cause any other immediate, objective disadvantages. Second, these highly-skilled immigrants should stand a high likelihood to find a job in a different country, too. As a result, this group is not objectively threatened (e.g., upcoming deportation; visa expiration). Observed negative post-vote experiences are thus likely the result of more subjective thoughts and feelings, which would demonstrate that social exclusion hurts even if the threat is primarily subjective in nature. For instance, highly-skilled immigrants might feel less welcome in the country, be worried about dependents and friends, or about not getting a future renewal of their residence permit. Going beyond the individual level, these immigrants might even experience vicarious ostracism due to identifying with the group of (German) immigrants or other highly-skilled immigrants and, thus, experience a threat against their ingroup [19–22].

A strong amount of research in social as well as political sciences has focused on why immigrants are excluded and discriminated against in societies (e.g., [23–26], see also [27]) and how structural variables or long-term discrimination affects immigrants’ well-being (e.g., [28–32]). Common to these lines of research is that social exclusion is understood as a general social disadvantage of immigrants over a long period of time [27]. Here we take a different perspective and focus on how immigrants with high social status and education who most likely do not experience discrimination on a daily basis experienced a singular exclusionary act, namely a popular vote of their host society. This perspective is inspired by research on social exclusion and ostracism, particularly by the temporal need-threat model of ostracism [7], which we further elaborate next.

### Social exclusion in the laboratory

The temporal need-threat model (of ostracism proposes that individuals are highly sensitive to the smallest threats of being excluded, rejected, or devalued by other humans, which has repeatedly been demonstrated in laboratory research on social exclusion (e.g., [4–9]). This sensitivity is rooted in humankind’s fundamental need to belong [33] and the vital necessity to become a member of cooperative social networks and groups. To avoid exclusion, humans have therefore developed a functional system that is able to detect even the slightest hints of rejection or exclusion [9–11]. Correspondingly, several laboratory studies have demonstrated that even minimal exclusionary threats, such as not receiving a ball in a virtual ball-throwing game, cause strong feelings of pain, decrease mood and threaten the four fundamental needs of belongingness, self-esteem, control, and meaningful existence [7, 12–15]. In the temporal need-threat model, this immediate negative reaction to exclusionary threat is termed the *reflexive stage*. Reflexive reactions have been shown to be very strong and robust across a variety of personality or situational characteristics, such as whether individuals were excluded by ingroup or outgroup members [4, 5, 13, 34], were socially anxious or not [35], or benefitted financially from being excluded [36].

Following the reflexive stage, individuals enter the *reflective stage*, in which they typically engage in behaviors aimed at restoring their needs, such as seeking for new connections [37] as well as distancing themselves from or even aggressing against the people who excluded them [38, 39]. Finally, if social exclusion is not resolved, individuals enter the so-called *resignation stage*. Here, impaired social connectedness which results from social exclusion has been connected to depression as well as general decreases in life satisfaction [7, 40, 41].

### Popular votes as exclusionary threats

To the present date, research on social exclusion, ostracism and the temporal need-threat model has been conducted mainly in the laboratory, focusing on interactions in small groups with clearly identifiable perpetrators and rather ambiguous situations [7]. However, in real life, exclusionary threats can occur in larger contexts as well: In fact, the term “ostracism” derives originally from the Greek practice of *ostrakismos*, which describes a popular vote in ancient Greece, by which a citizen would be expelled from the city of Athens for ten years [42]. In this tradition, we suggest that a popular vote on immigration regulation represents a real-life exclusionary threat, which is characterized by several specific aspects:

First, in the case of a popular vote, an individual is not threatened with exclusion because of some personal flaws, but because she or he possesses a specific group membership, namely a foreign citizenship [20, 43]. This qualification does not soften the blow of social exclusion, though: Individuals’ emotional experience is strongly dependent on the group that they identify with [44–46] and thus, exclusion because of group membership can even intensify experienced threat if this membership is permanent [20]. While immigrant status can change, the acquisition of Swiss citizenship is often highly restricted [47]. As a result, even second- or third generation immigrants might not be granted citizenship.

Second, a popular vote is a nation-wide political event with abstract perpetrators (i.e., an unidentified majority). The vote transforms this majority’s feelings, preferences, and implicit attitudes into hard numbers [48], so that immigrants receive statistically unambiguous feedback on how well they are accepted in the respective country. Accordingly, it is likely that current immigrants would perceive the result of the Swiss vote as a signal of not being welcome by the Swiss people and thus not accepted as a part of society (that is, being excluded).

Third, a vote on immigration regulation will eventually be transformed into a law. Even though the result does not expel current immigrants from Switzerland, the vote might have objective consequences on future immigrants, such that their residency or working requests might be rejected. While again, this might first and foremost affect low-skilled immigrants, driven by the success of the vote, other initiatives might form that may also aim to restrict current or highly-skilled immigrants’ rights and may be even harsher (as was recently the case in Switzerland, see the initiative “For the effective expulsion of foreign criminals” in 2016). The resulting uncertainty about what the results of the vote mean and which other initiatives might follow could add to the experience of exclusionary threat even for current, highly-skilled immigrants.

Based on these considerations, the temporal need threat model, and laboratory research on social exclusion [7], we derived predictions about how highly-skilled immigrants might have felt after the Swiss vote against mass immigration. It should be noted here, that highly-skilled immigrants may not identify with the category “immigrant” as a whole in everyday life. Instead, highly-skilled immigrants might generally feel more similar to Swiss than to culturally-remote immigrant groups (e.g., immigrants from Arab countries). But perceived membership of social categories is variable, and given the context of the vote, it appears likely that highly-skilled immigrants self-perceive themselves as belonging to the social group they formally belong to: immigrants.

### Immigrants’ affect following the vote: Hurt, threat, and negative mood

In line with the temporal need-threat model of ostracism [7], we assumed that immigrants would report having experienced negative affect after the vote, similar to negative reactions that occur immediately when being excluded in the laboratory (the so-called “reflexive stage”). More specifically, we assumed that immigrants had experienced feelings of hurt, negative mood, and threat to their fundamental needs of belonging, self-esteem, control, and meaningful existence when they had first heard about the results of the vote.

### Resilience / Vulnerability factors: Political orientation and social support

The temporal need threat model of ostracism assumes that the first negative reaction in the reflexive stage is relatively robust. However, research shows that despite their strong sensitivity for exclusionary threats, individuals do not react to all experiences of social exclusion in the same way (e.g., [49, 50], for an overview see [7]). The subjectivity of social exclusion experiences has especially been emphasized in more recent extensions of the temporal need-threat model [8] regarding the importance of socially situated cognitions, that is, how social exclusion situations are cognitively construed in the first place. Here, we focus on two factors which are directly related to the immigration debate and supposedly strongly influenced the situated construal of the popular vote for immigrants: a) one’s personal norms and attitudes regarding immigration, which are eventually related to one’s political orientation as well as b) social support from the Swiss people.

#### Anti-immigration attitudes and left/right orientation

Some social exclusion experiences can be attributed to situational norms or are in line with an individual’s personal value system [51]. Such experiences are not interpreted as a threat to one’s inclusionary status, and therefore hurt less than exclusion experiences which violate social or personal norms [8, 52–54]. To illustrate, previous studies have shown that left-wing participants who are excluded based on a gender quota perceived this exclusion as less threatening than right-wing participants [8]. Presumably this is because right-wing participants typically do not support such a left-wing political agenda and thus, exclusion due to gender quotas is seen as a norm violation and subjective threat. In contrast, exclusion due to gender quotas is consistent with left-wing individuals’ norms and thus experienced as less threatening. It is important to note here that strong norms and attitudes do not necessarily reflect individuals’ momentary social experience (that is, whether an individual is excluded or included) but exist independently. In other words, individuals react negatively to violations of their personal norms in general, no matter whether they are directly affected in the current situation. In the aforementioned study, right-wing individuals who were included to the discussion also reported more negative affect than included left-wing individuals, possibly due to the perceived norm violation [8].

In case of the Swiss vote, we expected accordingly that individuals’ norms about immigration in general would also predict their affect following the vote. More specifically, we assumed that a more left-wing orientation would predict a more negative affect following the vote and that this relation would be mediated via anti-immigration attitudes. This is because persons with a political left-wing orientation are more in favor of policies benefitting immigrants (e.g., [26, 55, 56]) and should accordingly tend to have positive attitudes and norms regarding immigration. The result of the vote therefore represents a norm violation, which is related to negative affect. In comparison, people with a more moderate or right-wing orientation are oftentimes more in favor of immigration regulation. Even though it might appear to be contrary to their self-interest, conservative immigrants might also have generalized personal norms and attitudes that are in line with immigration regulation, namely that a country should be allowed to protect its character and not let too many foreigners in. Since personal norms and attitudes create a feeling of obligation to uphold these attitudes and act in line with them [51], conservative immigrants might uphold these ideals even when the country that enforces them is not their country of origin [26] but rather their country of residence. As a result, immigrants with a more right-wing orientation should have reacted less negatively to the result of the vote.

#### Social support

Social support and the extent to which a person feels personally accepted and appreciated in her or his current surroundings can buffer an individual against the pain of social exclusion [29, 32, 57, 58]. Especially, previous contact with members of the outgroup is an effective buffer against threat [59]. We therefore hypothesized that support which derives from the same apparent majority that ostracized the individual, i.e., the Swiss people, would be positively associated with the affect following the vote. This feeling of support from the Swiss people can derive from at least two possible sources, namely a) the individual has many Swiss friends and b) the individual lives in a canton where the majority voted against the initiative. We assumed that both factors could offer structural as well as functional support and thereby reduce the negative consequences of exclusionary threat.

As for structural support, both the availability of Swiss friends as well as a canton vote that differed from the result of the general vote might prevent immigrants from overgeneralizing the result of the vote to the conclusion that they are excluded and disliked by “all of the Swiss people.” Instead, their friends or the result in their canton might point them to the fact that they were “only” excluded by a narrow majority of 50.3 which might (physically or socially) not even be in their immediate surroundings, as well as reinforce the impression that at least some Swiss people are on their side. As for Swiss friends, naturally this argument only holds if one’s friends have indeed voted against the initiative. However, intergroup contact hypothesis [60, 61], would suggest that Swiss citizens who befriend immigrants are less likely to be opposed to immigrants and immigration in general.

Regarding functional social support, it has been shown that excluded individuals typically have a heightened need for social reaffiliation with people they perceive as non-perpetrators [38, 62, 63]. There might be more reaffiliation options for individuals with many Swiss friends or individuals who live in an immigration-supporting canton than for individuals with few or no Swiss friends or immigrants living in immigration-opposing cantons.

### Consequences: Life satisfaction, attitude change towards the host country, and desire to leave

Following the first (reflexive) negative reaction to an exclusionary threat, in the reflective stage excluded individuals typically aim to cope with it by aiming to reaffiliate with others as well as try to disengage and distance themselves from the perpetrators [39], who are typically perceived as negative [38]. This negative view as well as distancing attempts should be stronger if the initial exclusionary threat was perceived as very severe. In addition, to avoid further contact with Swiss people and to increase chances to connect with other, non-Swiss people, an immigrant’s best option might be to leave the country. We therefore expected that more negative affect following the vote would be associated with a more negative attitude toward the excluding majority, that is, the Swiss people, as well as a stronger interest to leave the country in the future.

In laboratory research, induced exclusion experiences are minor and people usually recover within a couple of minutes. In the field, especially when unresolved, the initial negative effects of exclusionary threats continue to influence further behavioral and affective consequences. In the TNMT [7], this phase is called the *resignation stage* and characterized by signs of depression, decreased life satisfaction and learned helplessness. In line with this, previous research has shown that experiencing social exclusion in general negatively affects satisfaction with life for immigrants [e.g., 28, 30, 31, 32]. Accordingly, we expected that the reported affect following the vote is associated with life satisfaction insofar that the stronger the negative post-vote affect, the lower one’s life satisfaction. Note that life satisfaction judgments have been shown to be strongly affected by situational variation [64].

Taken together, we expected reported negative affect following the vote to predict a negative change in one’s self-reported attitude towards Switzerland as well as increased considerations to leave the country. All three aforementioned variables that measure potential long-term consequences to the vote (attitude change towards Switzerland, considerations to leave the country, and life satisfaction) are henceforth referred to as “outcome variables.”

### “Hot” and “cold” reactions to exclusionary threats

So far, we have focused on associations with the negative affect that immigrants experienced following the vote, a link henceforth described as the “hot path.” While this hot path corresponds to predictions of the temporal need-threat model and most laboratory research on social exclusion, in a real life setting such as the Swiss vote, there might be additional cognitive processes, which are independent from the affective experience of threat. For instance, individuals in favor of immigration might in general devalue any country that tightens immigration regulation, even if they do not feel personally threatened by a specific event such as the vote. This is especially likely in a sample of highly-skilled immigrants such as the one investigated here, where individuals may normatively dislike immigration regulation but at the same assume they will not be concerned by the result of the vote because they are needed in the country. To account for such processes, it therefore appeared plausible to assume a second, “cold path,” that is more cognitive-driven and not related to reactions to the actual vote. This distinction is further in line with a body of research that generally distinguishes between two judgmental processes, one mainly driven by (hot) affective responses and the other mainly driven by (cold) cognitions (e.g., [65, 66]).

In terms of our hypothesized model, the “hot path” links anti-immigration attitudes to the three outcome variables via reported post-vote affect. More specifically, we assumed that less anti-immigration attitudes would predict more negative affect following the vote. Negative affect is subsequently related to a more negative attitude change towards Switzerland, increased considerations to leave the country and impaired life satisfaction. The “cold path” is represented by a direct effect of anti-immigration attitudes on attitude change towards Switzerland and considerations to leave the country that is *not* mediated via affect following the vote. While attitude change and considerations to leave the country represent consequences that directly respond to one’s opinion about Switzerland, satisfaction with life refers more towards one’s own self and one’s subjective life conditions. This is why we assumed that the relation between anti-immigration attitudes and satisfaction with life should be fully mediated by affect following the vote.

## Hypothesized model

In summary, prior laboratory research on social exclusion allows for the conclusion that an event like the Swiss initiative against mass immigration may perceived as an exclusionary threat by immigrants in Switzerland. Against this background, we hypothesized that immigrants in Switzerland experience negative affect following the vote, and postulate that political orientation and social support would influence the strength of the reported affect in the following way:

H 1: The more left-wing, the less anti-immigration attitudes.
H 2a: The less anti-immigration attitudes, the more negative individuals’ affect following the vote.
H 2b: The higher the proportion of Swiss friends, the less negative individuals’ affect following the vote.
H 2c: Immigrants from cantons that have voted pro-initiative report more negative affect following the vote.
H 3: Left/right orientation is related to affect following the vote indirectly via anti-immigration attitudes.

Moreover, we assumed that the degree of the negative affect had a direct effect on all three outcome variables (hot path):

H 4a: The more negative the affect, following the vote, the more negative the attitude change towards one’s host country.
H 4b: The more negative the affect following the vote, the more negative one’s life satisfaction.
H 4c: The more negative the affect following the vote, the higher one’s desire to leave the country.

Finally, we assumed that one’s attitude would also directly influence the outcome variables (cold path):

H 5a: The stronger ones’ anti-immigration attitudes, the more positive the attitude change towards one’s host country.
H 5b: The stronger ones’ anti-immigration attitudes, the lower one’s desire to leave the country.

**Fig 1:**
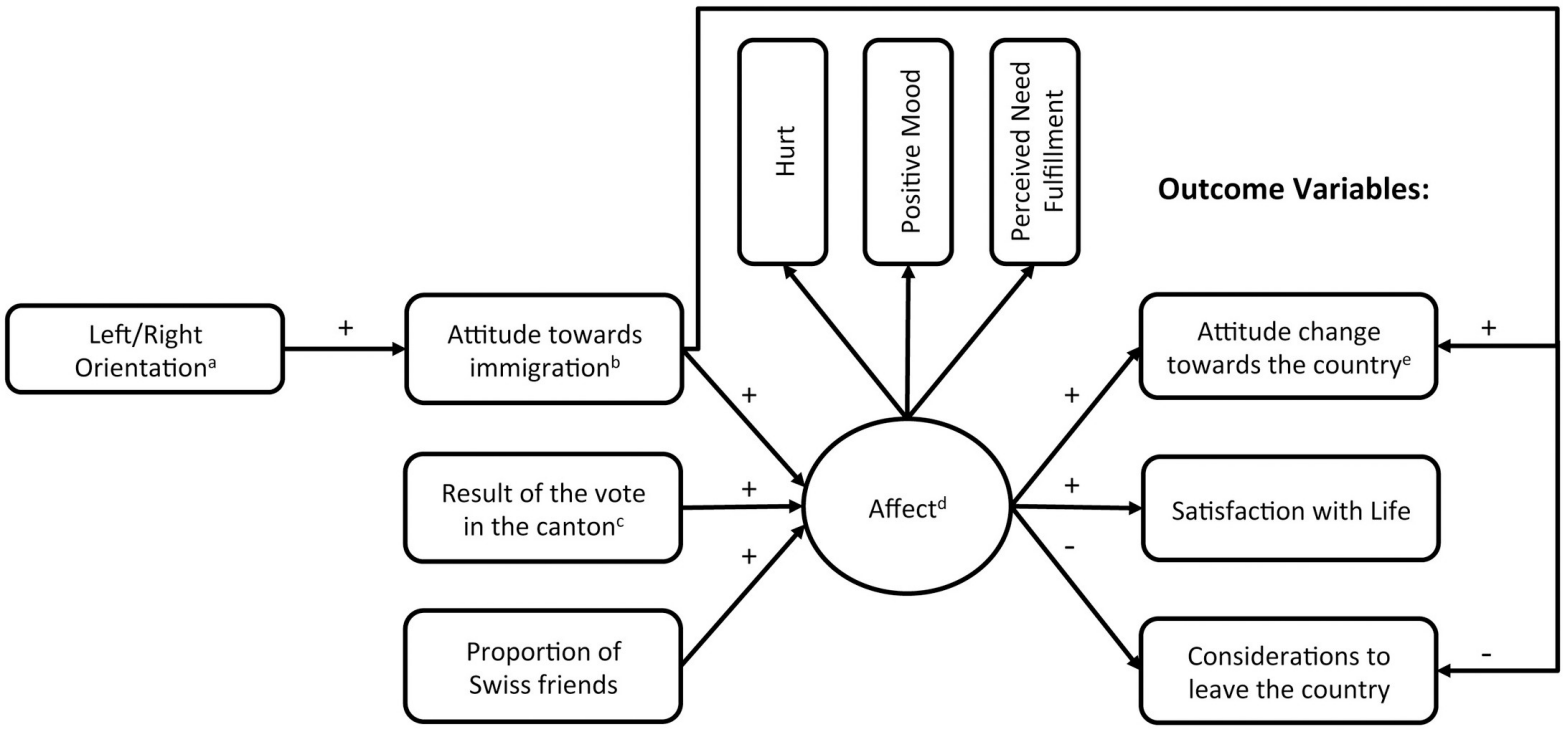
Hypothesized structural equation model. ^a^ high values indicate a right-wing orientation. ^b^ high values indicate strong anti-immigration attitudes. ^c^ 0 = pro Initiative, 1 = contra Initiative. ^d^ high values indicate positive affect following the vote. ^e^ low values indicate an attitude change in a negative, high values in a positive direction

All predictions were integrated into a hypothesized path model that is depicted as Fig 1.

## Method

### Participants

We conducted an online survey three weeks after the Swiss vote “Against mass immigration”in 2014 among German-speaking immigrants of Switzerland with a foreign citizenship, which was distributed via several mailing lists and online groups (e.g., Facebook groups, university mailing lists) as well as snowball sampling. Because of the timing, a unique dataset was acquired. In line with [67] the study was conducted in full accordance with the Ethical Guidelines of the Swiss Psychological Society (SGP-SSP) and the American Psychological Association (APA). By the time the data were acquired (March 2014) it was not customary at Basel University to seek ethics approval for survey studies. All questionnaires in the study were anonymous questionnaires and no identifying information was obtained from participants during the survey (after the survey, participants could enter their email address for a lottery; this data was stored separately from the survey data). Before starting the survey, participants were presented with a consent form stating explicitly that their participation was voluntary, that they may withdraw from the study at any time without explanation, and that the data is treated confidentially. Participants then gave informed consent via a yes/no item. Particularly, they confirmed that they were at least 18 years old, had read the consent form, and agreed to participate in the survey. Moreover, participants could easily withdraw from the study at any time by closing the Internet browser and further had the option to indicate that their data should not be used (yes/no item) at the end of the survey.

The survey included persons who were currently living, working, or studying in Switzerland, but did not hold Swiss citizenship. Overall, 332 participants finished the questionnaire, but seven participants did not want their data to be analyzed. Moreover, because some of the measures were specific for immigrants who live in Switzerland (canton vote, considerations to leave the country) we excluded cross-border commuters without a Swiss residence from the statistical analysis (43 participants). The following analyses are all based on 282 participants who currently had residence in Switzerland at the time of the vote (193 females, *M*_age_ = 33.60, *SD* = 8.58). Given the uncertainty regarding the root mean square error of approximation of the final model, it was not possible to conduct an exact a priori power analysis [68]. However, popular rules of thumb suggest to use at least 10 participants per variable in the final model (in our case = 100 participants) [69] or at least 10 participants per estimated parameter (in our case = 150 participants) [70]. These conventional criteria were exceeded by our final sample with the factor 2.82, respectively the factor 1.88.

Participants had lived in Switzerland between 1 month and 32 years (*M =* 6.11 years, *SD =* 6.15), with 34.0% holding the (permanent) settlement Permit C, 59.6% the (temporary) residence Permit B, and 3.9% the short-term residence Permit L. In the current sample, 89% of participants had successfully completed the academic track of secondary school and 70.6% possessed a university degree. Participants lived in 21 different cantons (out of 26), the most frequent ones being Zürich (29.4%), Basel-Stadt (26.6%), Aargau (9.2%), and Luzern (7.8%). There were 110 participants living in cantons who had supported the initiative and 172 participants living in cantons that had opposed the initiative. Most participants lived in cantons in which German is one of the official languages (97.8%) and, the major immigrant groups in the survey were Germans (71.7%) and Austrians (13%). This was not surprising since the language of the survey was German.

The present sample consists mainly of highly skilled immigrants from Germany and is therefore not representative for the entire immigrant population of Switzerland. However, Germans represent one of the major groups of Switzerland’s immigrant population [17, 18]. Moreover, the large proportion of academic background in the sample further equals the proportion reported in official statistical surveys for German immigrants in Switzerland (e.g., "Schweizer Arbeitskräfteerhebung"; [18]). Strictly speaking, our conclusions are refined to this group. We address the question of whether and how our findings would extend to different immigrant groups in the Discussion.

### Measurements

#### Predictors: Left/Right orientation and proportion of Swiss friends

To assess Left/Right orientation, participants were asked: *“Please indicate your political attitude on the following scale”* (semantic differential; *1 = left*, *11 = right*). To assess the proportional amount of Swiss friends, participants were asked: *“How many of your friends are Swiss*?*”* (*1 = none or almost none*, *7 = everybody or almost everybody*).

#### Mediators: Anti-immigration attitudes and affect following the vote

To assess anti-immigration attitudes, we created a four item scale (“*High immigration is more of a risk than a gain for a country*,*” “High immigration destroys the character of a country*,*” “Countries have to regulate their immigration to uphold their cultural identity*,*” “In the long run*, *high immigration has both economic as well as cultural disadvantages*,*”* 7-point Likert scales, *1 = not at all*, *7 = very much;* Cronbach’s α = .87).

Affect following the vote was assessed via three constructs: Need Fulfillment, Mood, and Hurt [7]. To make sure that participants’ affect was indeed related to the vote, participants were instructed to put themselves back in the situation when they had first heard about the result of the vote. Previous research has indicated that individuals can remember as well as relive social exclusion experiences very accurately [71]. For reasons of test efficiency, Need Fulfillment and Mood were assessed with short scales [8].

Specifically, Need Fulfillment was assessed with four 9-point semantic differentials representing four fundamental needs (adjectives: *rejected–accepted* (belongingness), *devalued–valued* (self-esteem), *powerless–powerful* (control), and *invisible–recognized* (meaningful existence), Cronbach’s α = .77). Mood was assessed with a single item measure (9-point semantic differential; *1 = bad*, *9 = good*). Hurt was assessed with two items (*“The result of the vote hurt me” and “The result of the vote disappointed me personally”*; 9-point Likert Scale; *1 = not at all*, *9 = very much*, *r* = .63, *p <* .001).

#### Outcome variables: Attitude change, considerations to leave the country, satisfaction with life

To assess attitude change towards Switzerland following the vote, participants were asked: *“The following question relates to your evaluation of Switzerland after the vote*. *Compared to my attitude before the vote*, *my attitude towards Switzerland became more…”* Participants then answered three 7-point semantic differentials (*worse–better*, *negative–positive*, *dissatisfied—satisfied*; Cronbach’s α = .93).

Considerations to leave the country at present were assessed with a single item: *“To what extent do you consider it an option to move away from Switzerland right now*?*”* (7-point scale, coded as *1 = not at all*, *7 = very much*).

To assess participants’ current satisfaction with life, we used the German translation of the *Satisfaction with Life scale* (Cronbach’s α = .87; [72]), which consists of five items, e.g., *“I am satisfied with my life*.*”*

### Analyses

The postulated path model reflecting the assumed associations (see Fig 1) was computed using structural equation modeling with MPLUS Version 7.1 [73]. We used the Maximum Likelihood estimator with robust standard errors (MLR) to conduct our analyses. While all other constructs were included as manifest variables, *affect following the vote* was modeled as a latent factor using the highly correlated variables *Hurt*, *Need Fulfillment*, and *Mood* as indicators (smallest *r* = -.63).

Following the suggestions of Hu and Bentler [74], we report the model fit using the χ^2^-test for model fit and a combination of misfit (SRMR, RMSEA) and fit indices (CFI). In line with the recommended rules of thumb for cut-off values by Schermelleh-Engel, Moosbrugger and Müller [75], we distinguish between an acceptable (*p ≥* .01, SRMR ≤ .10, RMSEA ≤ .08, CFI ≥.95) and a good model fit (*p* ≥.05, SRMR ≤ .05, RMSEA ≤ .05, CFI ≥.97).

## Results

### Descriptive results

In line with the literature, we state that the vote represents an exclusionary event and that immigrants had experienced negative affect following the vote. In the absence of a natural control group or a pre-vote message, it is not possible to test this premise with a comparative analysis. However, many of the variables were measured as semantic differentials (need fulfillment, mood, attitude towards Switzerland) and thus had a positive and a negative pole. Very cautiously, one may thus look at the group means descriptively in order to determine a general level within the sample.

On average, participants‘ reported affect when they first heard about the results of the vote tended to be negative (combined Need Fulfillment: *M =* 3.36, *SD =* 1.37; Mood: *M =* 3.24, *SD =* 1.72; Hurt: *M =* 5.84, *SD =* 2.42). For Need Fulfillment, 86 percent of the participants reported a value below the scale midpoint of 5.00 (72% for Mood), thus indicating a negative experience. Moreover, on average, participants reported that their attitude towards Switzerland had been impaired after the vote (*M =* 2.72, *SD =* 1.34), with 73 percent of the participants reporting values below the scale midpoint of 4.00. Variables without a direct reference to the vote generally reflected a more positive attitude (Considerations to leave the country: *M =* 2.70, *SD =* 2.04, Satisfaction with life: *M =* 4.95, *SD =* 1.19, on 7-point scales). Participants who held a temporary permit differed from participants with a permanent permit neither in their reported affect following the vote, nor in the level of attitude change towards Switzerland (all *p* > .165). The amount of time participants already lived in Switzerland did not affect these variables either (all *p* > .318).

As for the predictors, on average participants were left-wing (*M =* 4.71, *SD =* 1.76), had little anti-immigration attitudes (*M =* 2.72, *SD =* 1.34), and a rather small proportion of Swiss friends (*M =* 2.98, *SD =* 1.65). The zero-order correlations of all variables are displayed in Table 1.

**Table 1:** Zero order correlations.

	(1)	(2)	(3)	(4)	(5)	(6)	(7)	(8)	(9)
(1) Left/Right Orientation ^a^									
(2) Anti-Immigration Attitudes ^b^	.41**								
(3) Canton vote ^c^	-.08	-.08							
(4) Proportion of Swiss Friends	.04	.16**	-.11						
(5) Hurt	-.12*	-.32**	-.12	-.08					
(6) Positive Mood	.12*	.26**	.16**	.16**	-.63**				
(7) Need Fulfillment	.09	.31**	.11	.16**	-.64**	.68**			
(8) Attitude change^d^	.19**	.36**	.13*	.17**	-.55**	.61**	.57**		
(9) Life Satisfaction	.05	.06	.02	.15*	-.20**	.20**	.19**	.23**	
(10) Considerations to leave the country	-.09	-.24**	.05	-.32**	.27**	-.26**	-.24**	-.28**	-.30**

^a^ High values indicate a right-wing orientation.

^b^ High values indicate strong anti-immigration attitudes.

^c^ 0 = pro Initiative, 1 = contra Initiative

^d^ low values indicate an attitude change in a negative, high values in a positive direction

* p < .05

** p < .01.

### Structural equation modeling

**Fig 2:**
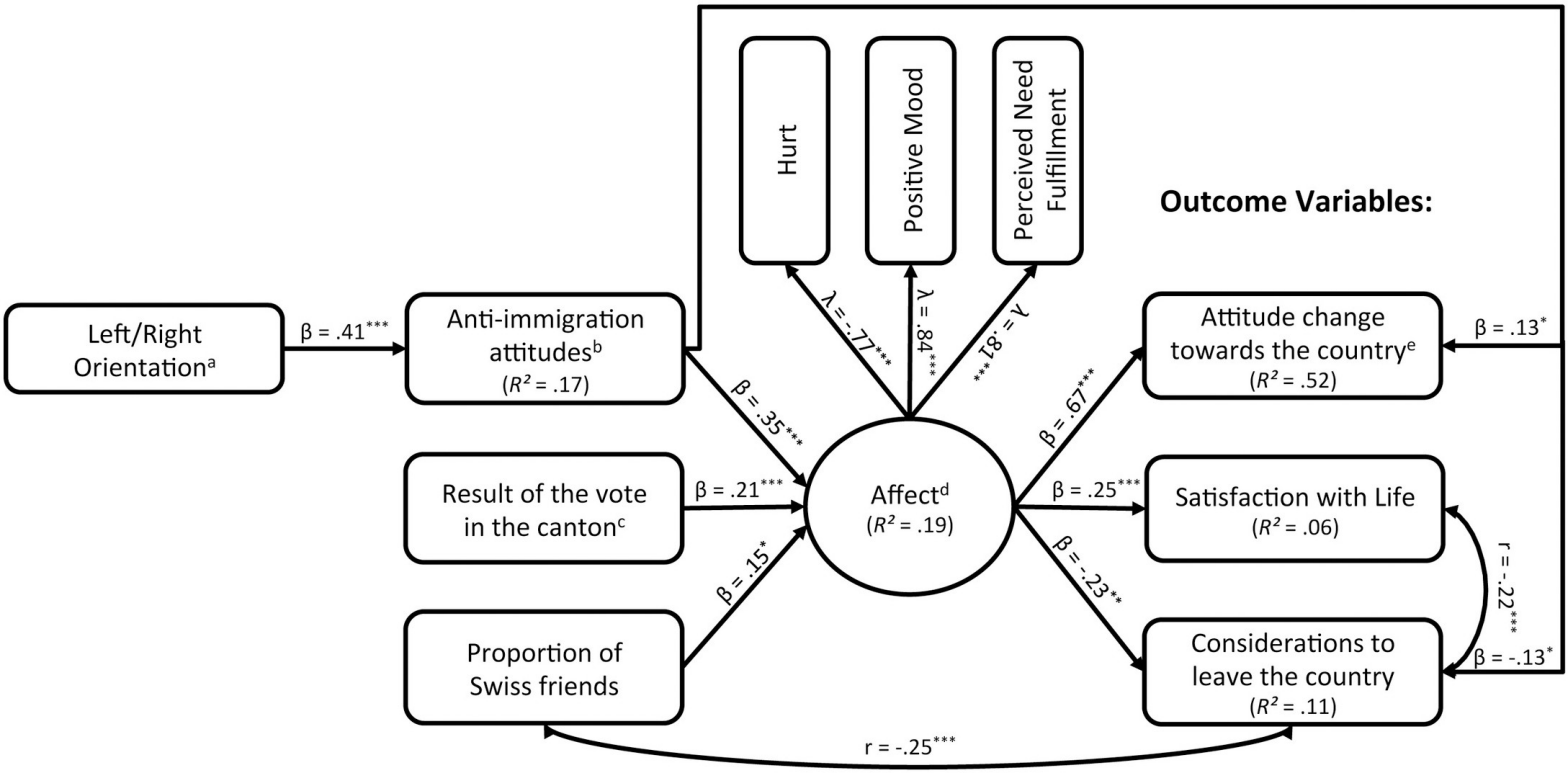
Observed structural equation model. Only significant correlations between the dependent variables are displayed (p < .05). ^a^ high values indicate a right-wing orientation. ^b^ high values indicate strong anti-immigration attitudes. ^c^ 0 = pro Initiative, 1 = contra Initiative. ^d^ high values indicate positive affect. ^e^ low values indicate an attitude change in a negative, high values in a positive direction

At first the postulated model showed an acceptable fit to the data (χ^2^ (27; *n* = 282) = 45.58, *p =* .014, *CFI* = .971, *RMSEA* = .049, *SRMR* = .048) with a power of .72 to assess the fit of the model (power analysis based on the RMSEA[68]). We computed modification indices to explore if there were further reasonable associations between model variables impairing the model fit in our initial model. These modification indices suggested an additional association between the proportion of Swiss friends and considerations to leave the country. Even though this association was not originally included in the hypothesized model, it appeared conceptually reasonable that the two variables should be connected. Besides the effects of current events like the vote on immigration regulation, people should be more likely to stay in places where they have established a social network. Since we had had no a priori hypothesis on this association, we freed an undirected effect between the two variables. After this step all fit indices improved, suggesting a good fit between our model and the data (χ^2^ (28; *n* = 282) = 32.38, *p =* .260, *CFI* = .993, *RMSEA* = .024, *SRMR* = .038) as well as a final power of .90. The final adjusted path model with the estimated path coefficients is shown in Fig 2.

In the following, we will focus on the observed path coefficients and their relation to our initial hypotheses. First of all, a more left-wing political orientation was linked to less anti-immigration attitudes *(H1)* and thereby indirectly associated with more negative affect following the vote (*H3;* see Table 2 for the indirect effect). Moreover, having many Swiss friends as well as living in a canton that had opposed the initiative predicted less negative affect following the vote (*H2b-c*). More negative affect following the vote was further associated with a more negative change of attitude towards Switzerland, as well as increased considerations to leave the country and decreased satisfaction with life (*H 4 a-c*).

**Table 2:** Expected indirect effects.

	β_indirect_	*z*	*p*
Left/Right Orientation → Anti-Immigration Attitudes → Affect	.14	4.22	< .001
Anti-Immigration Attitudes → Affect → Attitude change	.24	4.61	< .001
Anti-Immigration Attitudes → Affect → Life Satisfaction	.09	3.04	.002
Anti-Immigration Attitudes → Affect → Considerations to leave	-.08	-2.61	.009

As for the two postulated pathways, less anti-immigration attitudes directly predicted a more negative affect following the vote *(H2a)* and subsequently had an indirect effect on the three outcome variables, representing the postulated “affective/hot path” (*Table 2)*. As for the direct (cognitive/cold) path, less anti-immigration attitudes directly predicted a more negative attitude change and increased considerations to leave the country, unmediated by affect *(H5a&b)*.

## Discussion

This contribution presents a unique dataset collected three weeks after the Swiss popular vote “Against mass immigration”in 2014. In the present survey, a sample of highly-skilled, German-speaking immigrants reported having experienced feelings of hurt and negative mood as well as threat to their fundamental needs of belongingness, self-esteem, control, and meaningful existence following the Swiss vote against mass immigration. Moreover, the empirical evidence supports the assumption that living in immigration-opposing cantons, being politically left-wing and having few Swiss friends is related to a more negative affect following the vote. Negative affect further predicts a negative attitude change towards Switzerland, increased considerations to leave the country, and decreased life satisfaction.

### Effects of exclusionary popular votes

To our knowledge, the current study is the first to investigate affects and cognitions following a popular political vote against immigration on a subsample of the affected minority, namely highly-skilled immigrants. Results from the current study indicate that highly-skilled immigrants can feel threatened in the aftermath of such an exclusionary vote and provide important information about the negative consequences as well as possible factors that influence how such an experience is perceived.

Specifically, the results highlight the importance of subjective interpretations of exclusion experiences: First, the data suggest that exclusionary threats can vary considerably in their strength according to one’s own personal attitudes and norms, such as one’s political opinion. This result is in line with findings from prior experimental research, which show that if exclusion is consistent with social as well as one’s personal norms and attitudes, then negative affective reactions are attenuated or even dissipate altogether [8]. As a potential caveat, in should be noted that there might be variation in what participants thought about when they answered questions about “immigration attitudes,” especially whether they included themselves in that category or not. For instance, Helbling and colleagues [17] have argued that the stereotypical immigrant is culturally distant (e.g., Muslim), badly educated, and of low social status, which would not apply to highly-skilled persons from German-speaking countries. In contrast, Asbrock and colleagues [76] assume that the stereotype of an immigrant is defined by the major immigrant group in a specific region, which would then include (highly-skilled) Germans as the second-largest immigrant group in Switzerland. In any case, given the context of the vote against mass immigration in the present survey, we think that it is likely that especially participants with a high level of education would have understood that technically, they are immigrants themselves, even if they might not categorize themselves like that in everyday life. Nevertheless, we also believe that it may be an interesting endeavor to put our assumption to the test by including group identification in future research on the impact of popular votes on immigration restrictions.

Second, the negative affect that participants in our survey reported suggests that exclusionary threats do not need to be objective and concrete to concern an individual (e.g., an upcoming deportation or visa expiration). Even a mostly subjective threat such as a vote concerning future immigration of others might be related to considerable distress. Several underlying reasons may account for why participants felt threatened: feeling not welcome and disliked by one’s host country, experiencing an offense against one’s ingroup or vicarious ostracism [45, 77], concerns that family members might not be able to follow into the country which could leave the individual isolated in the future, general insecurity about what the result of the vote means and what concrete measures are to follow, or worries about how welcome one really is.

While we did not ask directly whether participants experienced the popular vote as an exclusionary threat, the typical low levels in the fundamental needs of belonging, self-esteem, control, and meaningful existence that are characteristic for social exclusion episodes is highly suggestive in this regard. Moreover, in a different study nine months later, we asked 326 participants about a similar popular vote (“Ecopop initiative”) in which feelings of being excluded and ignored were assessed pre- and post-vote *(*7-point semantic differentials, *excluded–included*, *ignored–acknowledged)*. In contrast to the initiative against mass immigration, the Ecopop initiative was opposed by the Swiss people, which resolved the exclusionary threat and prevented us from testing our hypotheses. Nevertheless, this data is partly interesting because participants reported stronger feelings of exclusion pre-vote (while the exclusionary threat was still in the air) compared to post-vote (when the exclusionary threat was resolved), *t*(325) = 17.94, *p* < .001 (*M*_*pre-vote*_ = 2.97, SD = 1.26; M_*post-vote*_ *=* 4.93, *SD =* 1.07). Moreover, immigrants reported significantly more feelings of exclusion than Swiss participants, *t*(218.43) = 4.43, *p* < .001 (*M =* 2.96, *SD =* 1.26 and *M =* 3.63, *SD =* 1.28, respectively). Given that the two votes were rather similar in content, these results suggest that participants in the present survey experienced the vote against mass immigration as some form of exclusionary threat, too.

An interesting question is whether the obtained results are specific for popular votes or would generalize to other demonstrations of public opinions or attitudes that can also be found in countries without a direct democracy (e.g., results of opinion polls, attention-drawing demonstrations against immigration, etc.). On the one hand, the effects of such demonstrations of public opinion are more subtle than a popular vote and usually do not involve direct consequences, which is why they might lead to less drastic decreases in immigrants’ affects. On the other hand, since they are more commonplace, negative effects might sum up over time and result in an overall feeling of not being welcome.

### Social exclusion beyond the laboratory

The results further extend previous research in the field of social exclusion, which was usually confined to laboratory studies that generally relied on small groups and visible, clearly identified perpetrators. While laboratory research is highly important to unravel and investigate the basic processes which underlie the experience of social exclusion, the utilized paradigms can merely represent an approximation to multi-faceted social exclusion situations in real life. Accordingly, it is of equally high importance to transfer obtained knowledge about variables and processes from the lab to the real world and apply them to natural, realistic settings such as the Swiss vote. The present study is a first step in that direction.

In the case of the Swiss vote, the source of exclusionary threat was an anonymous majority opinion which was not directed at the participant specifically. Still, participants reported having experienced threat and negative affect following the vote. That the perpetrators cannot be clearly identified might create additional uncertainty and threat, since individuals often may not know whether the concrete persons they are interacting with in everyday life belong to this majority or not.

Moreover, the temporal need-threat model and laboratory research on social exclusion have strongly emphasized the role of the primary affective reaction and the effect of different threatened needs such as belonging or control on behavioral outcomes [78]. However, laboratory studies usually focus on rather short timeframes up to one hour and minor exclusionary threats from which participants recover quickly. While the first affective reaction to exclusionary threat is certainly of high importance for immediate subsequent behavior, individuals who have time to consider their options for days or weeks might also behave in more cognitive, “coldly” processed ways that do not necessarily reflect the amount of threat and hurt that was experienced in the first moment. For instance, the final decision to leave the country might also highly depend on how immigrants perceive their job opportunities in Switzerland after the vote.

### Differences due to language and immigrants’ social status

Our survey mainly represents the experiences of highly skilled immigrants from German-speaking countries, who represent one of Switzerland’s major immigrant groups [17]. Most participants further lived in German-speaking Switzerland, which tends to be less immigration-friendly compared to the French-speaking cantons [55, 79]. In our investigated sample, experienced negative affect was a function of the canton vote, thus given that all of the French-speaking cantons voted against the initiative, immigrant groups from these cantons might have been affected less, or not at all.

Given the high level of education that participants in our survey reported, it is further important to discuss whether and how our results might transfer to immigrants with lower status or skill. First, immigrants who speak one of the Swiss languages (i.e., German, French, or Italian) fluently or even as their mother tongue might be able to follow the media coverage about the initiative more easily than immigrants who do not. This also applies to more educated immigrants, who might additionally also be more interested in the political affairs such as public votes. Accordingly, immigrants from countries such as Germany, Austria, France, and Italy as well as immigrants with higher status and education could have experienced more negative affect following the vote. Moreover, immigrants from EU countries, who so far have profited from the free movement of workers as part of the Schengen agreement, might experience the vote as a (norm) violation of the Schengen agreement; until the vote, the Schengen agreement gave EU citizens the right to apply for work in Switzerland without being subjected to quotas. This change in rights could have resulted in a stronger affective reaction of EU citizens compared to immigrants from third party countries.

Finally, one could argue that high-status immigrants have less experience with stigmatization and discrimination in everyday life than do low-status immigrants. This might have resulted in a higher level of surprise about the result of the vote compared to low-status immigrants. However, expecting exclusion usually does not affect the initial pain that results from an exclusion experience [8, 80]. In general, laboratory research on social exclusion has demonstrated that especially the initial negative effect on affective measures that is associated with exclusionary threat is strong, robust, and shows relatively little variation with regard to interpersonal or intergroup differences [33, 63, 81]. There are substantial differences in behavioral reactions to surprising and expected social exclusion, however: Whereas surprising exclusion seems to elicit stronger aggressive responses [82], especially regularly occurring and thus expected episodes of long-term exclusion have been connected to feelings of depression, alienation, and helplessness [83].

Thus, it remains unclear and up to future research to investigate whether high and low status immigrants would experience the same initial pain and negative affect when they learned about the result of the vote. In the long run, it is likely that low and high status immigrants would react very differently once the initial “shock” about the vote result has worn off. On average, highly-skilled immigrants might have better social buffers such as more power, a better social network, as well as more alternatives than lowly-skilled immigrants. For instance, highly skilled immigrants from EU countries might have better options to return to their home country, and therefore disengage and discard the result of the vote more quickly. Low status immigrants, in contrast, might experience more devastating long-term consequences as a result. Such differential questions might be interesting to pursue in future studies including diverse groups of immigrants.

### Limitations

All our conclusions are drawn based on cross-sectional data, which is not sufficient to clearly address issues of causality. For instance, one could assume that immigrants experience strong negative affect in general, independent of the result of one specific vote. Such relations are partially reflected in the cold path, or more specifically, in the direct link between anti-immigration attitudes and considerations to leave the country that is not mediated via affect following the vote. However, there are several reasons why one might argue for some causal effect of the vote on the investigated variables:

First, our results are in line with predictions of the temporal need-threat model of ostracism [7], which proposes a causal path of reflexive reactions (immediate feelings of threat and pain) resulting in subsequent reflective reactions (e.g., attempts to distance oneself from the perpetrators). Additionally, all of the underlying process assumptions are backed up by the rich experimental findings of social exclusion research that allow for causal conclusions (for an overview, see [7]).

Second, the variables measuring affect and attitude change towards Switzerland explicitly referred to participants’ subjective experience *in the context of the vote*. In the absence of a pre-vote measure or a comparable control group that was not subjected to exclusionary threat, we looked at the magnitude of these variables, and found them to be closer to the negative pole of the semantic differentials than to the positive pole. This was not the case for variables that were not directly related to the vote, however, such as considerations to leave the country and satisfaction with life. Thus, it appears unlikely that participants’ responses regarding negative affect and attitude change following the vote reflect a general negative attitude but rather appear to be linked to the vote.

Third, the canton where the immigrants lived is an objective variable that is directly related to the voting results. The finding that immigrants from cantons that supported the initiative experienced more negative affect and more negative attitude change following the vote than immigrants from cantons that opposed the initiative further supports the assumption that it was indeed the vote which was responsible for these effects. It should be mentioned though, that the cantons that had opposed and supported the initiative most likely differ on other criteria than the mere result of their vote. For instance, rural cantons had mainly supported the initiative, whereas more urban cantons such as Basel-Stadt, Zürich, and Geneva, that also have the largest proportion of immigrants [16], had opposed the initiative. As a result, immigrants living in initiative-opposing cantons might have better social networks and feel generally more accepted in their everyday life even prior to the vote. It is therefore possible that individuals do not react to the canton vote per se, but that the canton vote is an indicator of a more or less immigration-friendly climate in general. Thus, it would be interesting for future field studies on the effects of social rejection on immigrants to additionally investigate general effects of a possible hostile climate on the well-being of immigrants. This would help to distinguish the solitary effects of political decisions and popular votes from those effects that are situated in the general political climate.

### Long-term consequences

The current survey focuses on affect and cognition shortly after the vote, which makes it difficult to derive assumptions about long-term behavioral consequences. For instance, it remains an open question whether considerations to leave the country would actually transform into actions and affect moving behavior. However, there is evidence that Swiss voting results such as from a vote against minarets can affect moving behavior and make immigrants refrain from moving into communities where they assume not to be welcome [84]. Interestingly and in line with the assumptions presented here, this effect was not confined to Muslim immigrants and also is stronger among highly-skilled compared to lowly-skilled immigrants.

Our study was not conducted immediately after the vote but three weeks later. Still, participant’s reported affect following the vote was linked to their current satisfaction with life. Since the result of the vote was extensively discussed in the Swiss media even weeks and months after the ballot, it is quite possible that even one single vote might result in enduring consequences. Previous research which has linked social exclusion to both a decreased satisfaction with life as well as to symptoms of depression [7, 85], further stresses the notion of how powerful exclusionary threats are and that even short-term reactions to exclusion should not be treated lightly.

## Conclusion

In this contribution, we investigate affect and cognitions of Swiss immigrants following a powerful exclusionary threat, namely a popular votes on immigration. Even though it is unclear whether immigrants will be objectively excluded, highly-skilled immigrants of Switzerland reported subjective feelings of threat and hurt following the vote. These feelings are subsequently related to decreased satisfaction with life as well as a lower affiliation with one’s host country.

## Supporting information

S1 File Survey Raw Data. (DAT)

S2 File SPSS Syntax for Data Preparation. (SPS)

S3 File Prepared Data for MPLUS Analysis. (DAT)

S4 File MPLUS Syntax for Structural Equation Modeling. (INP)
