# Correction: Under threat by popular vote: German-speaking immigrants’ affect and cognitions following the Swiss vote against mass immigration

**DOI:** 10.1371/journal.pone.0182703

**Published:** 2017-08-02

**Authors:** Selma Carolin Rudert, Stefan Janke, Rainer Greifeneder

## Notice of Republication

This article was republished on April 26, 2017, as an incorrect version of S1 File was published in error. Please download this article again to view the correct version.
